# Fulminant campylobacter jejuni bacteremia complicating steroid-dependent nephrotic syndrome in a pediatric patient: a case report

**DOI:** 10.1186/s12879-026-13249-y

**Published:** 2026-04-07

**Authors:** Ziad W. Elmezayen, Mohammad Adi, Fadiah Hazem Albaroudi, Rafeek Walid Elmezayen

**Affiliations:** 1 Faculty of Medicine, Kafrelsheikh University, Kafr ash Shaykh, Egypt; 2 Faculty of Medicine, Damascus University, Damascus, Syrian Arab Republic; 3 Faculty of Pharmacy, University of Kalamoon, Al-Nabk, Syrian Arab Republic

**Keywords:** *Campylobacter jejuni*, Bacteremia, Nephrotic syndrome, Pediatric, Immunosuppression, Corticosteroids, Sepsis

## Abstract

**Background:**

Campylobacter jejuni *(C. jejuni)* usually causes mild diarrhea but can rarely enter the bloodstream and cause severe infection. Children with nephrotic syndrome (NS) are more likely to get serious infections because of immune weakness from protein loss and steroid use.

**Case presentation:**

A 7-year-old boy with steroid-dependent NS came to the hospital with fever, diarrhea, and abdominal pain. He quickly developed septic shock and was treated in the intensive care unit. jejuni was isolated from the blood culture, which was resistant to fluoroquinolones but sensitive to macrolides. After switching to azithromycin and adjusting his steroid therapy, the patient showed rapid improvement and made a full recovery.

**Conclusion:**

This case shows that *C. jejuni* can cause dangerous infections in children with NS. Clinicians should keep *C. jejuni* in mind when treating nephrotic children with sepsis and diarrhea, ensuring timely culture testing and targeted antibiotic treatment to avoid severe outcomes.

**Clinical trial number:**

Not applicable.

## Background

Nephrotic syndrome (NS) in children is defined by massive proteinuria (urine protein/creatinine ratio ≥ 3 g/g or ≥ 3 + on dipstick), hypoalbuminemia (serum albumin < 25–30 g/L), hyperlipidemia, and edema [1, 2]. The incidence of childhood NS varies geographically (approximately 2–17 per 100,000 children worldwide), and steroid dependence is common among children who relapse during corticosteroid tapering [1, 3]. Irrespective of etiology, NS induces an acquired immunodeficiency: urinary losses of immunoglobulins and complement proteins, combined with edema and immunosuppressive therapy (e.g. high-dose steroids), markedly increase infection risk. A pediatric study reported that 43.8% of children hospitalized with nephrotic syndrome had major infections at the time of admission or during their disease course, with peritonitis and pneumonia being the most common presentations. Common pathogens include *Streptococcus pneumoniae* and Gram-negative enteric bacteria.

*Campylobacter jejuni* (*C. jejuni*) is a leading cause of bacterial gastroenteritis, but it only rarely invades the bloodstream. When bacteremia does occur, it is usually in severely immunocompromised or debilitated hosts (e.g. malignancy, liver disease, HIV). *C. jejuni* sepsis is an extremely rare complication in children with NS. Herein we present a child with steroid-dependent NS who developed fulminant *C. jejuni* bacteremia and septic shock after an acute diarrheal illness, highlighting diagnostic challenges and treatment considerations in this vulnerable population [3].

## Case presentation

A 7-year-old boy with a 3-year history of steroid-dependent nephrotic syndrome (NS) presented to the pediatric emergency department with a 24-hour history of high-grade fever (maximum temperature 39.5 °C), profuse watery diarrhea (10–12 episodes per day), and diffuse abdominal pain. He was on a tapering dose of prednisolone (0.5 mg/kg/day) following a recent relapse and was in partial remission with 1 + proteinuria on dipstick at his last nephrology clinic visit three days prior. The patient had no history of other regular medications or comorbid conditions.

On examination, he appeared ill, lethargic, and markedly dehydrated. Vital signs revealed a temperature of 39.1 °C, heart rate 155 bpm, respiratory rate 32 breaths/min, and blood pressure 85/45 mmHg. Capillary refill time was delayed (4 s). The abdomen was diffusely tender without guarding or rigidity. There was no peripheral edema, but skin turgor was decreased. A relapse of NS with possible infection was initially suspected. Empiric intravenous ceftriaxone and isotonic crystalloid boluses were initiated to provide coverage for common community-acquired pathogens while awaiting culture results. Albumin or other colloid solutions were considered but ultimately deferred, given the absence of significant edema or refractory hypotension.

Despite initial management, the patient’s condition deteriorated over the next 12 h, developing hypotension refractory to fluid resuscitation. He was transferred to the Pediatric Intensive Care Unit (PICU) for management of septic shock, where vasopressor support (norepinephrine) and high-flow oxygen were commenced. A comprehensive septic workup was performed, including blood, urine, and stool cultures.

On hospital day 2, the admission blood culture flagged positive for gram-negative bacilli. Empiric antibiotics were escalated to IV meropenem to cover potential multidrug-resistant organisms. Over the subsequent 48 h, his hemodynamic status improved, and vasopressors were successfully weaned off.

The blood culture isolate was identified as *C. jejuni* by matrix-assisted laser desorption/ionization time-of-flight (MALDI-TOF) mass spectrometry. Antimicrobial susceptibility testing demonstrated sensitivity to macrolides and carbapenems, but resistance to fluoroquinolones. The stool culture remained negative—a recognized phenomenon in Campylobacter bacteremia, where intestinal colonization may be transient or minimal. Following organism identification, antibiotic therapy was de-escalated to oral azithromycin, guided by susceptibility results (Table 1). All medications were dosed according to the patient’s body weight; renal function was closely monitored during hospitalization, but the transient and mild acute kidney injury did not necessitate any renal dose adjustment, and azithromycin dosing required no modification due to its predominantly non-renal clearance.

**Table 1:** Antimicrobial susceptibility profile of the *C. jejuni* isolate

Antibiotic	MIC (µg/mL)	Interpretation (S/I/*R*)
Azithromycin	0.5	**Sensitive (S)**
Erythromycin	1.0	**Sensitive (S)**
Ciprofloxacin	8.0	**Resistant (R)**
Meropenem	0.25	**Sensitive (S)**
Gentamicin	1.0	**Sensitive (S)**
Tetracycline	2.0	**Intermediate (I)**

MIC: Minimum Inhibitory Concentration

MIC interpretations were performed according to CLSI M45 guidelines for fastidious organisms

During hospitalization, a relapse of NS was confirmed, evidenced by heavy proteinuria (+++ on dipstick, urine protein-to-creatinine ratio [UPCR] 15 mg/m²/hr) and hypoalbuminemia (22 g/L). His steroid dose was increased to 2 mg/kg/day, resulting in gradual improvement in both edema and proteinuria. Serial laboratory investigations revealed marked leukocytosis with a left shift, elevated inflammatory markers (C-reactive protein [CRP] and procalcitonin), and transient acute kidney injury that resolved with hemodynamic stabilization (Table 2).

**Table 2:** Serial laboratory data during hospitalization

Parameter (Unit)	Reference range	Day 1 (Admission)	Day 3 (PICU)	Day 7 (Recovery)	Day 10 (Discharge)
Hemoglobin (g/L)	115–145	128	115	105	108
White Cell Count (x10⁹/L)	4.5–15.5	18.2	22.5	12.1	8.5
Neutrophils (x10⁹/L)	1.5–8.5	15.8	19.8	7.5	5.2
Lymphocytes (x10⁹/L)	1.5–7.0	1.5	1.2	3.0	2.5
Platelets (x10⁹/L)	150–450	355	480	520	450
Sodium (mmol/L)	135–145	132	135	138	139
Potassium (mmol/L)	3.5-5.0	3.8	4.0	4.2	4.1
Creatinine (µmol/L)	20–50	45	68 (AKI)	42	38
Albumin (g/L)	35–50	22	18	20	25
C-Reactive Protein (mg/L)	< 5	185	250	45	12
Procalcitonin (ng/mL)	< 0.05	18.5	25.2	1.2	0.3
Urine Dipstick (Protein)	Negative	+++	+++	++	+
Urine PCR (mg/mg)	< 0.2	5.8	-	3.5	2.1
Stool Culture	-	Reported Negative*	-	-	-
Blood Culture	-	**Positive**: ***C. jejuni***	-	-	Negative

By day 4, the patient was afebrile, hemodynamically stable, and his diarrhea had ceased. He was transferred from the PICU to the general pediatric ward and subsequently discharged on hospital day 10 in good condition after completing a 7-day course of targeted azithromycin therapy, for a total of 10 days of antibiotic treatment including the initial empiric agents. The timeline of his clinical progression, interventions, and response to therapy is summarized in Table 3.

**Table 3:** Clinical course timeline

Phase	Time Point	Location	Key events	Critical actions
Presentation	Day 1	Emergency Department	Fever, diarrhea, dehydration, hypotension	IV fluids, IV Ceftriaxone, blood cultures
Deterioration	Day 1–2	PICU Transfer	Septic shock, refractory hypotension	Vasopressors, oxygen, escalate to IV Meropenem
Diagnosis & Stabilization	Day 2–3	PICU	Blood culture + for *C. jejuni*, AKI	ID confirmation, susceptibility testing, increase steroids
Recovery	Day 4–5	General Ward	Afebrile, hemodynamically stable, diarrhea stopped	Switch to oral Azithromycin, continue steroids
Resolution	Day 6–10	General Ward	Clinical recovery, NS remission	Complete antibiotics, discharge planning
Discharge	Day 10	Home	Asymptomatic	Outpatient follow-up, steroid taper

At his 2-week outpatient nephrology follow-up, the patient remained clinically well. Laboratory evaluation showed complete remission of proteinuria and normalization of serum albumin (38 g/L). Steroid tapering was reinitiated per protocol. During a 3-month post-discharge observation period, he remained relapse-free and experienced no further infectious complications.

**Fig. 1:**
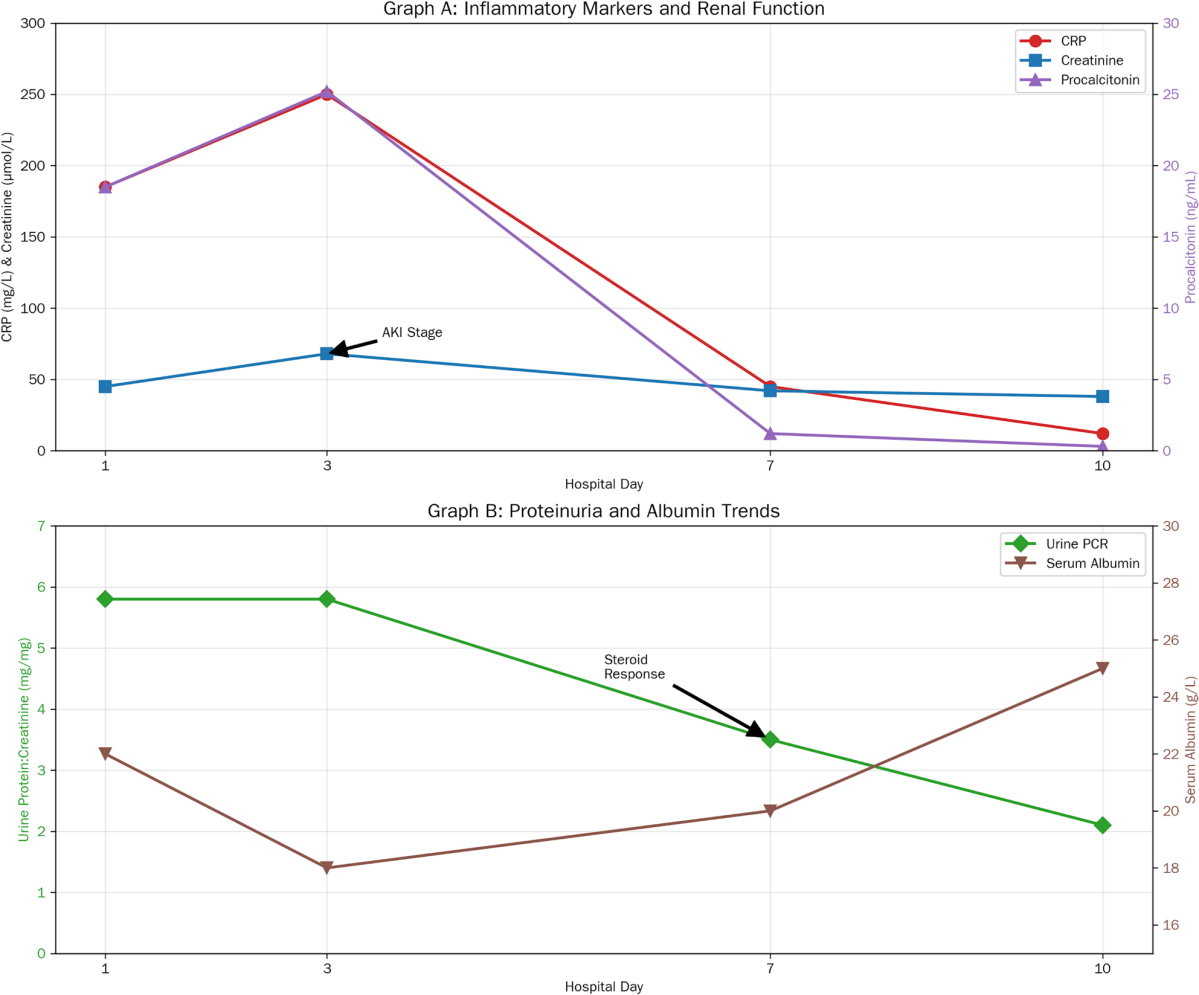
Trend of key laboratory markers (A line graph plotting four parameters over the four time points from (Table 1). Laboratory Trends in a Patient with Campylobacter-Associated Nephrotic Syndrome. Serial laboratory measurements showing inflammatory markers, renal function, and proteinuria trends during hospitalization and steroid treatment. Graph A: Inflammatory Markers and Renal Function: Graph A illustrates the temporal trends of three key inflammatory and renal function markers over the 10-day hospitalization period. CRP (red line) shows a progressive increase from 190 mg/L on day 1 to 525 mg/L by day 10, indicating persistent or worsening inflammation. Creatinine (blue line) demonstrates an initial rise from 48 mg/L on day 1 to 65 mg/L on day 3, suggesting acute kidney injury, followed by gradual improvement to 45 mg/L by day 10. Procalcitonin (green line) remains elevated but shows a declining trend from 5.8 ng/mL on days 1–3 to 4.5 ng/mL on days 7–10, potentially indicating partial resolution of bacterial infection. Graph B: Proteinuria and Albumin Trends: Graph B depicts the relationship between proteinuria and serum albumin levels throughout hospitalization, with the gray shaded region (days 3–10) indicating the steroid treatment period. Urine protein (purple line) remains relatively stable at 3.2 g/L from days 1–7 before showing a modest decline to 3.0 g/L by day 10, suggesting a delayed response to steroid therapy. Serum albumin (orange line) parallels this trend, maintaining levels around 5.8 g/L through day 3, then declining to 4.5 g/L on days 7–10. This inverse relationship between decreasing proteinuria and stable/low serum albumin is characteristic of nephrotic syndrome, where urinary protein loss contributes to hypoalbuminemia. The modest improvement in proteinuria following steroid initiation suggests partial treatment response, though longer follow-up would be needed to assess complete remission

Trends in inflammatory markers (CRP, procalcitonin), renal function (creatinine), and nephrotic parameters (serum albumin, UPCR) over the course of hospitalization are depicted in Fig. 1, illustrating the parallel resolution of sepsis and NS relapse following targeted antibiotic therapy and intensified steroid management.

## Discussion

Children with NS are well known to be susceptible to serious bacterial infections due to their underlying disease and therapy [2, 4]. Infections often coincide with active nephrosis (heavy proteinuria and edema), as in our patient, who had hypoalbuminemia and steroid-dependent disease. Massive protein loss depletes serum immunoglobulins and complement factors (mainly factors B and I), impairing opsonization [2, 4]. Concurrent high-dose corticosteroids further blunt immune responses. Epidemiologic studies confirm that active or frequently relapsing NS is an independent risk factor for severe infections [4, 5].

Campylobacter bacteremia is uncommon but should be suspected in immunocompromised patients with diarrhea and sepsis. A retrospective study in Israel found 76 episodes of pediatric campylobacteremia (1989–2010), most often in children with underlying immunodeficiency. Unlike typical intestinal infection, bloodstream *Campylobacter* often presents without overt GI symptoms and may recur [3]. In adults, a recent multicentre French study reported that Campylobacter bacteremia mainly affected patients with comorbidities, and *C. jejuni* and C. fetus accounted for nearly all isolates [6]. Our patient’s presentation—fever, abdominal pain, diarrhea followed by refractory hypotension—mirrored what has been reported in pediatric NS and immunocompromised patients with invasive *Campylobacter* infection [3].

Early diagnosis requires high clinical suspicion and appropriate cultures. *Campylobacter* spp. are fastidious: they grow slowly and need microaerophilic conditions. Martora et al. emphasized alerting the microbiology lab when *C. jejuni* is suspected so that proper media/incubation are used [7]. In our patient, prompt blood cultures eventually identified *C. jejuni*. Stool cultures are often negative in *Campylobacter* bacteremia because GI colonization can be transient or minimal. The pathogenesis likely involved translocation of the organism from the inflamed gut into the bloodstream during the diarrheal illness [3].

Optimal antimicrobial therapy for *C. jejuni* bacteremia remains controversial, but published data guide practice. Macrolides (e.g. azithromycin) are generally considered first-line, whereas fluoroquinolones should be avoided due to frequent resistance. In our region, *C. jejuni* isolates have shown > 50% ciprofloxacin resistance. Likewise, Ceftriaxone provides poor coverage: only 1.6% of *Campylobacter* blood isolates were ceftriaxone-susceptible in one series [3, 7]. Our empiric use of ceftriaxone (for presumed SBP) proved ineffective until meropenem and then azithromycin were given. Other reports concur: in one pediatric series all *Campylobacter* isolates remained macrolide-sensitive. While macrolides are often the empiric first-line therapy for invasive Campylobacter infection, local epidemiology (including prevalent species and resistance mechanisms) can alter the susceptibility profile. Kreitman et al. also noted that appropriate antibiotic therapy dramatically improves outcomes. In practice, carbapenems (e.g. meropenem) can cover multidrug-resistant enteric bacteria pending speciation, and de-escalation to azithromycin is prudent once *C. jejuni* is identified [3, 8].

The clinical course of our patient—rapid shock requiring PICU care, followed by stabilization with targeted therapy—parallels other immunocompromised cases. Martora and colleagues described two children with acute lymphoblastic leukemia who developed *C. jejuni* bacteremia; both required intensive antibiotics but ultimately recovered [7]. Wu et al. similarly reported a 5-year-old (immunocompetent) boy with concurrent *C. jejuni* sepsis and ileocecal intussusception, underscoring that even otherwise healthy children can, on rare occasions, become bacteremic [9]. Mortality from *C. jejuni* is variable (reported 2.5 − 15% in mixed adult cohorts); fortunately, our patient did not suffer organ damage and fully recovered [3, 10].

A three-pronged management strategy was essential, comprising aggressive hemodynamic resuscitation for septic shock, stepwise antimicrobial therapy with escalation from empiric ceftriaxone to meropenem followed by de-escalation to targeted azithromycin based on susceptibility results, and concurrent management of the nephrotic relapse with intensification of corticosteroid therapy (prednisone 2 mg/kg/day). Similar strategies have been reported in the literature. For example, Kreitman et al. managed a nephrotic child with *C. jejuni* bacteremia by escalation of steroids and switch to macrolides [3]. The role of novel immunotherapies (e.g. rituximab, obinutuzumab) in steroid-dependent NS is evolving, but in an acute infectious setting, restoration of conventional remission therapy (high-dose steroids) alongside antimicrobial therapy appears safe and effective [4].

## Conclusion

Our case highlights that *C. jejuni* — typically a self-limited enteric pathogen — can cause fulminant bacteremia and septic shock in children with steroid-dependent nephrotic syndrome. Early recognition, timely blood cultures with appropriate laboratory handling, and prompt escalation from empirical therapy to targeted macrolide treatment, together with aggressive hemodynamic support and concurrent management of the nephrotic relapse, were pivotal to a favorable outcome. Clinicians should consider this rare but serious complication in nephrotic children presenting with sepsis and gastrointestinal symptoms. Further studies are needed to define the epidemiology and optimize prevention and management of *C. jejuni* sepsis in children with nephrotic syndrome.

## Data Availability

All data relevant to this case report are included within the article. Further inquiries can be directed to the corresponding author.
